# Reduced opioids after total joint replacement surgery (REPAIRS): a pilot randomized controlled trial

**DOI:** 10.1186/s13018-025-06193-1

**Published:** 2025-08-20

**Authors:** Zhiwei Yang, The REPAIRS Pilot Trial Team

**Affiliations:** Institute for Musculoskeletal Health, Level 10N, King George V Building, Missenden Road, Sydney, PO Box M179, NSW 2050 Australia

**Keywords:** Total hip replacement, Total knee replacement, Opioids, Feasibility, Acceptability

## Abstract

**Background:**

Opioid analgesics are commonly used for controlling pain after total knee or hip replacement. However, it is unclear how much opioid should be prescribed at discharge after total hip and knee replacement surgery to optimize pain management but minimise adverse events. To determine the acceptability and feasibility of a trial comparing a reduced and standard opioid regimen following discharge from total knee or hip replacement.

**Methods:**

This pilot randomized controlled trial recruited participants from the pre-admission clinic of a metropolitan hospital in Sydney. All participants received a non-opioid regimen of naproxen 500 mg 12 hourly for 7 days, and paracetamol 1000 mg 6 hourly daily for 14 days. Participants were randomly allocated to the ‘standard’ group (oxycodone 5 mg taken as needed, maximum recommended frequency of 4 hourly, 20 tablets dispensed) or the ‘reduced’ group (received half the amount of oxycodone, taken half as frequently) at hospital discharge. Primary outcomes were screening to recruitment rate, percentage completing follow-ups, and acceptability of the trial procedures. Acceptability was measured quantitatively at weeks 1 and 2 post-discharge and qualitatively by interview at 6 weeks post-discharge. Adverse events were collected up to 6-weeks post-discharge.

**Results:**

In four months, we screened 254 people, of which 72 were eligible and 53 were recruited (recruitment rate = 21%). 93% participants completed all their follow up surveys. Participants scored the trial intervention between neutral and acceptable (3.6/5 for both groups and weeks). 39 interviews were conducted where both clinicians and patients reported strong support for the pilot trial design. Few adverse events occurred across both groups, the most common being constipation.

**Conclusions:**

This pilot trial indicated that a full-scale trial would be feasible and acceptable. Such a trial could provide evidence of comparative effectiveness for the reduced opioid regimen in this population.

**Trial registration:**

The trial was pre-registered in Australian New Zealand Clinical Trials Registry (ACTRN12623001070628).

**Supplementary Information:**

The online version contains supplementary material available at 10.1186/s13018-025-06193-1.

## Introduction

Opioid overuse is an enormous public health concern, with a key driver being the over prescribing of opioids after surgery [1, 2]. The role of opioids for pain management at discharge after surgery is being questioned by a recent systematic review which showed opioid analgesics caused more adverse events, but not more pain reduction, compared to non-opioid analgesics [3]. A limitation of the review is that it did not find any trials including populations undergoing major surgeries, such as total knee or hip replacement.

Total knee or hip replacement surgery is effective for treating severe knee or hip osteoarthritis [4, 5]. The demand for total joint replacement is high. In 2023, around 80,000 total knee replacements and 60,000 total hip replacements surgeries were conducted in Australia [6]. In the United States, approximately 800,000 knees and 550,000 hips are replaced annually [7].

Approximately 80–90% of the people undergoing total knee and hip replacement procedures are prescribed opioid analgesics for pain after surgery [8]. There is little direct evidence supporting the type, duration and dose of opioid that should be prescribed after hospital discharge. A recent meta-analysis of trials comparing opioids provided at higher doses or durations versus lower doses or durations at surgical discharge found no differences for pain control or other important outcomes [9]. However, available trials test relatively high amounts of prescribed opioids even in their low opioid-dose arms (e.g. 20 to 30 tablets of oxycodone 5 mg or Tramadol 50 mg). Awareness of limiting opioid use has increased since the publication of these trials. It is possible that patients may experience adequate pain relief from even lower amounts of opioids, if supported by a robust regimen of non-opioid analgesics. Dispensing only the amount of opioids that a person will require can serve to minimize adverse events such as constipation, dizziness, increased risk of long term use and dependence, and reduce the reservoir of unused opioids in the community, which can be diverted and/or misused [10].

A high-quality trial is necessary to fill the gap regarding whether lower total doses prescribed at discharge can provide satisfactory pain control, compared with the hospital standard opioid prescriptions after total knee or hip replacements [11]. To determine whether such a trial can be done, and, if so, how it can be done, our aim was to conduct a pilot randomized controlled trial to determine whether such a trial is acceptable and feasible for both clinicians and patients.

## Methods

The current pilot trial (REPAIRS) was approved by the Royal Prince Alfred Hospital’s Research Ethics Committee (NO.: 2023/ETH012940). The trial was pre-registered in Australian New Zealand Clinical Trials Registry (ACTRN12623001070628). The trial report was presented in line with the Consolidated Standards of Reporting Trials (CONSORT) 2010 statement: extension to randomized pilot and feasibility trials [12]. The trial proposal was presented for consumer feedback at the Australia and New Zealand Musculoskeletal Trials Consumer Advisory Group on the 11th of May 2023 and consumer feedback was incorporated in the final protocol.

### Design and participants

The REPAIRS trial was a pilot, parallel, 2-group, randomized controlled trial with participants allocated 1:1 to a reduced opioid regimen or a standard opioid regimen on top of a robust non-opioid regimen (details below), after discharge from total knee and hip replacement surgery. Participants were recruited from the pre-admission clinic of a public hospital in Sydney, New South Wales. This hospital conducts one of the largest volumes of joint replacement in Australia (approximately 700 procedures per year). Potential participants were provided with detailed study information, screened against the eligibility criteria by a medical doctor, and signed an informed consent form. Typically, participants presented to the preadmission clinic 1–3 weeks prior to their scheduled surgery. Inclusion and exclusion criteria are listed below:

#### Inclusion criteria

- Adults (age≥ 18 years) undergoing unilateral total knee or hip replacement.
- English proficiency required to complete questionnaires.

#### Exclusion criteria

- Contraindications for opioids (e.g. abuse history, allergy).
- Contraindications for non-steroidal anti-inflammatory drugs (e.g. renal, allergy gastrointestinal or peptic ulcer issues).
- Contraindications for paracetamol (e.g. allergy or liver disease).
- Participants who reported taking more than 15 Oral Morphine Milligram Equivalents (OMME(website-based calculator: https://www.mdcalc.com/calc/10170/morphine-milligram-equivalents-mme-calculator)) per day for more than 5 consecutive days within the previous 30 days.

### Randomisation

A randomisation schedule was generated a priori using randomly-permuted blocks of size 2 and 4 through a computer-generated method, stratified by surgery type (knee or hip). An independent researcher created the randomisation schedule and provided the group allocation in sealed, opaque and sequentially-numbered envelopes. At discharge, each participant was assigned an ID number corresponding to the next envelope in the series, which was opened on the discharge day to reveal the group allocation. Then the corresponding trial regimen for each group was prescribed by the doctor and dispensed by the pharmacy department after the doctor’s prescription.

### Sample size

We aimed to recruit 50 participants. The sample size was chosen based on pragmatic factors, including available funding and expected recruitment at the hospital. Sample size calculation was not performed as the trial purpose was feasibility testing rather than hypothesis testing.

### Intervention and control

All participants received a non-opioid regimen to take regularly comprising a 7-day supply of naproxen 500 mg taken 12-hourly, and a 14-day supply for paracetamol 1000 mg taken 6-hourly, to a maximum of 4000 mg per 24-hour period. Additionally, in the standard group, participants received oxycodone 5 mg, taken as required with a recommended frequency of 4 hourly, up to 6 times daily (20 tablets provided). A lower frequency approach was preferred over a lower dose approach, as providing < 5 mg of oxycodone would potentially be sub-therapeutic. Thus, in the reduced group, participants received oxycodone 5 mgs, taken as required with a recommended maximum frequency of 8 hourly, up to 3 times daily (10 tablets provided).

Both groups were given the same instructions on how to take the medicines. Additionally, we did not restrict participants access to non-study pain medication and healthcare services during the trial, and these were self-reported in trial surveys at each timepoint (week 1, 2 &6). An emergency hotline was set up to connect participants with a trial doctor in case they needed to access more medication and were unable to see their general practitioner.

### Outcome measurement

The primary focus was the feasibility and acceptability outcomes. We recorded the screening to recruitment rate, the percentage completing follow up surveys and the acceptability of the trial procedures. Recruitment was defined as the number of people who went on to be randomized and join the trial. The screening to recruitment rate was calculated by dividing the number recruited by the number screened. The percentage of participants completing follow-ups was calculated at weeks 1, 2 and 6. The reasons for unsuccessful recruiting were documented. Acceptability of the trial intervention was measured by the 4 item Acceptability of Intervention Measure (AIM) at week 1 and week 2. Each item of AIM included selections (Completely disagree, Disagree, Neither agree nor disagree, Agree, Completely agree) on a 5-point Likert scale (1 = completely disagree; 5 = completely agree) [13]. After completing the final survey at week 6, acceptability was also assessed by semi-structured interviews for both participants and relevant staff. Acceptability was defined as the degree to which participants and clinicians endorsed the trial design, interventions, and procedures of the pilot trial.

The secondary outcomes were collected via Redcap survey included pain severity (Numeric Pain Rating Scale (NPRS)) and quality of life (Eq. 5D-5 L), and adverse events collected at week 1, 2 and 6, as well as physical function (Knee Injury and Osteoarthritis Outcome Score (KOOS), The Oxford Knee Score (OKS), Hip Disability and Osteoarthritis Outcome Score (HOOS), The Oxford Hip Score (OHS)), collected at week 6. Adverse events were collected by asking participants “Have you experienced any adverse events, a new medical condition or a worsening of an existing medical condition since starting the study treatment for example dizziness or sweating?”. The NPRS needs individual to select an integer number between 0 and 10, which reflects individual’s current pain severity (0 = no pain; 10 = worst possible pain) [14]. The Eq. 5D-5 L comprises five dimensions and each dimension has five response levels. All answers from Eq. 5D-5 L are calculated according to the Australian value sets [15].

The KOOS and HOOS contain 42 items and 40 items, respectively, and both include five categories and are rated on a scale of 0 to 100 (0 being the worst function and 100 being the best function) [16, 17]. Both the OKS and OHS contain 12 items, and each item can be rated from 0 to 4. Therefore, the total score can range from 0 to 48, with 0 reflecting the worst function and 48 reflecting the best function [18, 19]. The number of oxycodone 5 mg tablets taken was collected by self-report in daily pain medicine diaries for the first 14 days post discharge.

### Statistical analysis

A statistical analysis plan was agreed upon by all authors before the start of analysis. The analysis plan also uploaded on Open Science Forum (https://osf.io/rxchu/). SAS^®^ version 9.4 was used to conduct quantitative data analysis. Confidence intervals were set as 95% and P-values were reported for completeness. All data from the trial were analyzed by an intention-to-treat approach.

In the qualitative analysis of acceptability, we aimed to explore whether there was support for the research question and trial design, as well as barriers and enablers that helped or hindered the pilot trial and could inform the design of a full-scale trial and implementation strategies. Semi-structured interviews were conducted by a trial research assistant, after pilot testing the interview guide with guidance from an experienced qualitative researcher (CJ) (Appendix 1). Interviews were recorded, transcribed verbatim, and thematically analysed using NVivo (version 14.24.0). Interview questions and coding of responses was loosely based on the Consolidated Framework for Implementation Research to assist in developing an optimal protocol for a future full scale trial [20]. Interview questions were designed to help trial staff understand the clinicians’ and participants’ views on the characteristics of the intervention, the characteristics of individuals, the inner setting, the outer setting, and the process. Interviews were coded by an investigator (ZY) and checked by a second investigator (CJ) afterward. Codes were then collapsed into key themes that covered the constructs of the Framework. A linear mixed model was conducted for quantitative comparison of acceptability between groups at week 1 and 2.

For each secondary outcome collected over multiple time points (weeks 1, 2 and 6), a linear mixed model was used to compare the two treatment arms. The baseline measurement was included in each model to provide adjusted mean effects. A compound symmetry correlation structure was applied to model the correlation between each measurement per participants. Adverse event was categorized by the ICD-10 code and serious adverse event was reported descriptively [21]. Adverse events, healthcare services, and pain medications were reported descriptively, and between-group differences were analyzed using Fisher’s exact tests.

## Results

### Participants flow

**Fig. 1:**
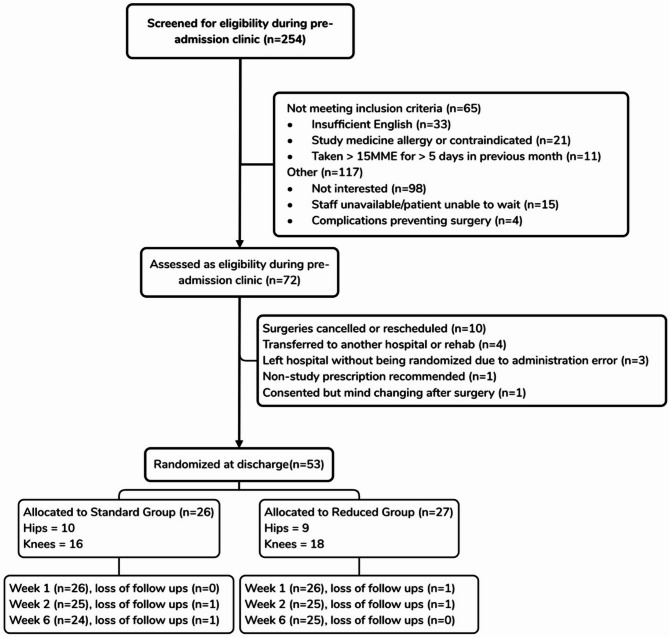
CONSORT 2010 flow diagram

254 potential participants were screened between 15th Jan and 9th May 2024, of which 72 were deemed eligible and consented. The most common reason for not being recruited was lack of interest in joining a trial, due to the burden of completing surveys (Fig. 1). 53 participants were randomized to the reduced group (*n* = 27; hip = 9; knee = 18) or the standard group (*n* = 26; hip = 10; knee = 16). The 19 eligible and consenting participants who were not randomized were excluded most commonly due to surgeries being cancelled or rescheduled (Fig. 1). No participant withdrew from either group at week 1. One participant dropped out of each group in week 2, and another participant withdrew from each group in week 6.

### Baseline characteristics

Baseline characteristics were mostly balanced at baseline, except participants in the standard group reported more pain in their knee or hip, and worse hip function (Table 1).

**Table 1:** Participants baseline characteristics

	Standard group (*n* = 26)	Reduced group (*n* = 27)
Age at preadmission (years) (mean (SD), N)	66.7 (8.5), 26	69.7 (8.0), 27
Sex (Female) (n/N (%))	14/26 (53.8%)	12/27 (44.4%)
Operated joint: Hip (n/N (%))	10/26 (38.5%)	9/27 (33.3%)
Operated joint: Knee (n/N (%))	16/26 (61.5%)	18/27 (66.7%)
BMI (kg/m^2^) (mean (SD), N)	30.2 (5.9), 23	26.8 (4.3), 26
***Ancestries***		
United Kingdom/European/Australian&New Zealander/Others* (n/N (%))	14 (53.8%) / 6 (23.1%) / 8 (30.8%) / 3 (11.5%)	16 (59.3%) / 8 (29.6%) / 9 (33.3%) / 1 (3.7%)
***Health insurance status***		
None/Private hospital and ancillary (extras)/Chose not to answer (n/N (%))	21 (80.8%) / 5 (19.2%) / 0 (0.0%)	23 (85.2%) / 2 (7.4%) / 2 (7.4%)
***Previous health services (last 7 days)***		
General practitioner/Imaging/Pathology (n/N (%))	4 (15.4%) / 8 (30.8%) / 2 (7.7%)	4 (14.8%) / 5 (18.5%) / 3 (11.1%)
***Previous pain medication (last 7 days)***		
Paracetamol/Non-steroidal anti-inflammatories/ Opioid alone or in combination with paracetamol (< 15 oral MME for < 5 days as per inclusion criteria) (n/N (%))	14 (53.8%) / 9 (34.6%) / 3 (11.5%)	13 (48.1%) / 7 (25.9%) / 2 (7.4%)
Pain severity (NPRS) (mean (SD), N)	6.3 (2.3), 26	5.6 (2.3), 27
***Function and quality of life***		
KOOS-12/ OKS (mean (SD), N)	45.3 (17.7) / 24.8 (9.2)	45.1 (17.0) / 26.3 (9.4)
HOOS-12/ OHS (mean (SD), N)	33.1 (16.7) / 19.8 (10.5)	42.4 (20.5) / 23.6 (11.5)
Quality of life (Eq. 5D-5 L) (mean (SD))	0.6 (0.3)	0.7 (0.3)

SD = standard deviation; N & n = number; BMI = body mass index; MME = morphine milligram equivalents; NPRS = numeric pain rating scale; KOOS = knee injury and osteoarthritis outcome score; OKS = oxford knee score; HOOS = hip dysfunction and osteoarthritis outcome score; OHS = oxford hip score; Eq. 5D-5 L = EuroQoL five-dimensional instrument the 5-level version

### Primary outcomes

The screening to recruitment rate was 21% over the 4-month recruitment period (254 screened and 53 recruited) (Fig. 1). At weeks 1, 2 and 6, the percentage completing follow-ups survey among all participants was 98%, 94% and 93%, respectively (Table 2).

**Table 2:** Completeness of baseline and follow-ups surveys by timepoint

Timepoints	Standard group (*n* = 26)	Reduced group (*n* = 27)	Total (*n* = 53)
Baseline (n (%))	26 (100%)	27 (100%)	53 (100%)
Week 1 (n (%))	26 (100%)	26 (96.3%)	52 (98.1%)
Week 2 (n (%))	25 (96.2%)	25 (92.6%)	50 (94.3%)
Week 6 (n (%))	25 (96.2%)	24 (88.9%)	49 (92.5%)

Participants scored the acceptability of the trial intervention on average between ‘neutral’ and ‘agree’ (3.6/5 in both groups in week 1, and 3.6/5 in both groups in week 2 on the AIM). There was no important difference for acceptability between groups (Appendix 2). Around 85% of participants from both groups showed ‘neutral’, ‘agree’ and ‘completely agree’ on the trial intervention (Appendix 2).

#### Qualitative measurement for acceptability

All participants and trial staff were invited to join the interview. 34 participants and 5 trial staff (one research assistant, two junior medicine officers and two orthopedic surgeons) accepted invitations for a semi-structured interview.

Overall, there was strong support for the research question from both participants and staff. Participants reported that the trial intervention was acceptable, because they felt the intervention amount is enough to start with after discharge and more prescriptions and other healthcare services could be accessed if required. Some participants were hesitant to take any opioids at all (i.e. were planning to only take the non-opioid components of the trial regimens) whereas others were expecting to require the opioids. These attitudes differed based on how each person viewed their personal level of risk (e.g. belief that the serious risks are not relevant to them as they are ‘not likely’ not become addicted). For example, one participant said, “I don’t like using pain killers … unless it’s necessary.’’ There was a strong theme that many participants wanted to avoid strong pain medicines like opioids, if they could manage their pain in other ways. Participants were very interested to know the results of the trial, e.g. if using less opioids could achieve adequate pain management. In rare cases, participants reported they might have difficulty accessing the healthcare service (such as to be prescribed more pain medicines in the event that their pain was unmanaged) because they lived alone or outside of metro areas.

Trial staff reported some minor concerns that the trial protocol that impacted feasibility, such as that they were unavailable at times for screening potential participants due to being occupied during preadmission clinic, which led to loss of potential participants. All specific themes and supporting quotes are provided in detail in Appendix 3.

### Secondary outcomes

There were no differences between groups for pain severity and quality of life at week 1. At week 2, there was a difference between groups in pain severity, with the reduced group reporting worse pain (adjusted mean difference − 1.4, 95% CI -2.58 to -0.26, *p* = 0.02). At week 6, participants who had a hip replacement in the reduced group had worse hip function than the standard group at week 6 (adjusted mean difference 18.3, 95% CI 2.41 to 34.11, *p* = 0.03). There were no differences between groups on pain severity, knee function and quality of life at week 6 (Table 3).

**Table 3:** Baseline adjusted average scores and treatment comparisons for pain severity, physical function and quality of life

		Adjusted mean (SE), *n*		Adjusted mean difference (95% CI), *p*-value
Score	week	Standard group	Reduced group	
Pain severity (NPRS)^1^	1	4.0 (0.4), 26	5.1 (0.4), 26	-1.1 (-2.21 to 00.04), *p* = 0.06
	2	3.5 (0.4), 24	4.9 (0.4), 24	-1.4 (-2.58 to -0.26), *p* = 0.02*
	6	3.0 (0.4), 23	3.9 (0.4), 22	-0.9 (-2.04 to 00.35), *p* = 0.16
Quality of life – Eq. 5D-5L^1^	1	0.8 (0.04), 26	0.8 (0.03), 27	-0.02 (-0.12 to 0.07), *p* = 0.63
	2	0.9 (0.03), 26	0.9 (0.02), 27	-0.003 (-0.07 to 0.07), *p* = 0.92
	6	0.9 (0.01), 26	0.9 (0.03), 27	0.05 (-0.03 to 0.12), *p* = 0.21
KOOS^2^	6	68.3 (4.0), 15	69.7 (4.0), 16	-1.4 (-12.8 to 10.13), *p* = 0.81
OKS^2^	6	33.7 (2.2), 15	35.6 (2.2), 15	-1.9 (-8.31 to 4.49), *p* = 0.55
HOOS^2^	6	81.1 (5.0), 9	62.9 (5.3), 8	18.3 (2.41 to 34.11), *p* = 0.03*
OHS^2^	6	40.0 (2.5), 9	32.9 (2.6), 8	6.6 (-1.25 to 14.37), *p* = 0.09

SE = standard error; NPRS = numeric pain rating scale; Eq. 5D-5 L = EuroQoL five-dimensional instrument the 5-level version. KOOS = Knee Injury and Osteoarthritis Outcome Score; OKS = Oxford Knee Score; HOOS = Hip disability and Osteoarthritis Outcome Score; OHS = Oxford Hip Score; ^1^Linear mixed model accounting for correlated observations due to repeated measures with adjustment for baseline score; ^2^Linear model adjusting for baseline score; * significant at type I error = 5%

#### Opioid consumption

The median number of tablets taken in the standard group was 9 (Interquartile Range (IQR) = 4 to 16) in week one and 1 (IQR = 0 to 8) in week two (Table 4). The median number of tablets taken in the reduced group was 9 (IQR = 3 to 14) in week one and 0 (IQR = 0 to 4) in week two (Table 4). Approximately 23% of participants in both groups took zero opioids during week 1, around half of participants took zero opioids in week 2. Both groups took approximately the same number of tablets.

**Table 4:** Opioid consumption at week 1 and 2 measured as number of oxycodone 5 mg tablets taken

Group	Week	Median	Mean	Standard Deviation	Q1	Q3
**Standard group**	1	9	10.6	9.4	4	16
	2	1	4.8	6.3	0	8
**Reduced group**	1	9	9.2	7.9	3	14
	2	0	5.2	8.7	0	4

#### Serious and non-serious adverse events

One serious adverse event occurred in the standard group involving a participant being readmitted to the hospital due to an issue with the surgical wound. Constipation was the most common adverse event reported in both groups. Table 5 shows the adverse events during follow-up; there were 15 and 20 adverse events in the reduced and standard groups, respectively (Table 5). There were no important differences between groups on pain medication use and healthcare services and their subgroups during follow-up (Table 5).

**Table 5:** Adverse events, healthcare service and pain medication

	Standard group(*n* = 26)	Reduced group (*n* = 27)	Between-group comparison
Adverse events at week 1, 2, or 6			
	20 EVT 14 PAT (53.8%)	15 EVT 13 PAT (48.1%)	Fishers exact p-value = 0.79
Healthcare service at week 1, 2 or 6			
Physio	28 EVT 18 PAT (69.2%)	25 EVT 18 PAT (66.7%)	Fishers exact p-value = 1.00
Imaging	5 EVT 4 PAT (15.4%)	3 EVT 3 PAT (11.1%)	Fishers exact p-value = 0.7
GP	18 EVT 13 PAT (50.0%)	21 EVT 17 PAT (63.0%)	Fishers exact p-value = 0.41
Other	7 EVT 6 PAT (23.1%)	9 EVT 8 PAT (29.6%)	Fishers exact p-value = 0.76
Pain medication at week 1,2 or 6			
Paracetamol	29 EVT 17 PAT (65.4%)	26 EVT 16 PAT (59.3%)	Fishers exact p-value = 0.78
Non-steroidal	10 EVT 7 PAT (26.9%)	13 EVT 7 PAT (25.9%)	Fishers exact p-value = 1.00
Opioid	15 EVT 11 PAT (42.3%)	10 EVT 8 PAT (29.6%)	Fishers exact p-value = 0.40
Opioid/paracetamol	1 EVT 1 PAT (3.8%)	5 EVT 4 PAT (14.8%)	Fishers exact p-value = 0.35
Other	1 EVT 1 PAT (3.8%)	6 EVT 6 PAT (22.2%)	Fishers exact p-value = 0.10

EVT = events; PAT = patients

## Discussion

Outcomes from the REPAIRS pilot trial support the feasibility and acceptability of trial to comparing a reduced amount of opioids with a standard amount, in addition to non-opioid analgesia, after discharge from total knee or hip replacement surgery. Overall, the pilot trial was welcomed by clinicians and participants. The resolution of uncertainty regarding what amount of opioids is required after discharge from total knee or hip replacement appears to be a major concern for both clinicians and participants.

Our screening to recruitment rate (21%) is comparable to recent similar trials, which range from 13 to 33% [22–24]. One important reason for the lack of participation among eligible participants was their unwillingness to join the project during pre-admission, and staff availability to assess potential participants in a busy clinical setting. In a fully funded trial, offering participants reimbursement for the time taken completing surveys (in this pilot trial we offered partial reimbursement due to limited funding), and employing dedicated trial staff for trial-related activities (including identifying and following up with potentially eligible participants) may help to overcome these issues.

It is clear from our qualitative investigation that both participants and clinicians supported this research question, and described a need for more evidence to guide decision-making around how to use opioids after major surgery like total knee/hip replacement. Of note, participants were willing to participate in a trial where they may be randomly assigned to take less opioids. This is in line with investigations in populations undergoing other types of surgery, such as plastic surgery, where less than one third wanted to take opioids after surgery [25]. REPAIRS participants were happy to be potentially randomized to the reduced post-discharge opioid prescription, citing the ability to access more if required, e.g. by seeing their general practitioner. Yet, participants who lived outside of metro areas reported that they would like to have more opioids dispensed at discharge due to concern they would have difficulty accessing other healthcare services. This might indicate a need for more access to general practitioner and/or pharmacy services in regional and remote areas, e.g. via telehealth. Despite this, no participants used our emergency hotline to obtain medication during the trial, including participants who lived outside of metro areas.

According to self-reported opioid consumption, both groups used a median of 9 tablets in the first week. Moreover, around a quarter of participants from both groups reported taking zero opioids in week 1, and around half took zero in week 2. This shows that people are likely being overprescribed opioids in current practice, such as in our standard care arm where 20 tablets were dispensed and less than half were used. Our reduced arm with 10 tablets appears to be closer to what patients in this pilot cohort actually used, but the large proportions of people using less (even zero) indicate that it is possible that even less opioids may be required to achieve satisfactory pain control.

One limitation is that the trial was unblinded, which could introduce bias such as performance bias and nocebo effect [26–30]. Those in the reduced group were aware that they were given less opioid tablets compared to standard care, and therefore may be influenced by their expectation of experiencing higher pain. Of note both groups reported taking similar amounts of opioids in Weeks 1 and 2. Another limitation is the small sample size in this pilot trial. Secondary outcomes between groups regarding pain severity, physical function and quality of life were imprecise and will need to be further explored in a fully powered trial [31, 32], including an assessment of the long term recovery on outcomes including pain and function. We emphasise that the main aim of this trial was to assess feasibility and acceptability, and not to compare treatment effectiveness. Also, given the small sample size, comparisons of adverse events were exploratory. These data provide an initial safety check that can inform the design of a full-scale trial.

In conclusion, this pilot trial has found that a trial investigating a reduced versus a standard regimen of opioids prescribed to people, in addition to a non-opioid analgesic regimen, at discharge after total knee and hip replacement is feasible and acceptable to both patients and clinicians. A full-scale randomized controlled trial, with minor modifications to overcome the problem points identified in this pilot, could inform whether opioid use can be reduced without negatively impacting patient outcomes.

## Supplementary Information

Below is the link to the electronic supplementary material.

Supplementary Material 1

Supplementary Material 2

## Data Availability

The datasets used and/or analysed during the current study are available from the corresponding author on reasonable request.

## Abbreviations

AIM: Acceptability of Intervention Measure
NPRS: Numeric Pain Rating Scale
KOOS: Knee Injury and Osteoarthritis Outcome Score
OKS: The Oxford Knee Score
HOOS: Hip Disability and Osteoarthritis Outcome Score
OHS: The Oxford Hip Score
IQR: Interquartile Range
