# Correction: Reduced opioids after total joint replacement surgery (REPAIRS): a pilot randomized controlled trial

**DOI:** 10.1186/s13018-025-06314-w

**Published:** 2025-09-27

**Authors:** Zhiwei Yang, The REPAIRS Pilot Trial Team

**Affiliations:** Institute for Musculoskeletal Health, Level 10N, King George V Building, Missenden Road, PO Box M179, Sydney, NSW 2050 Australia

**Correction to: Journal of Orthopaedic Surgery and Research (2025) 20:774** 10.1186/s13018-025-06193-1

In this article [1], The REPAIRS Pilot Trial Team should have been listed as the author group, comprising 11 members as a team.

The original article has been corrected.
