# Supplementary material for: Reduced opioids after total joint replacement surgery (REPAIRS): a pilot randomized controlled trial
Source: J Orthop Surg Res. 2025 Aug 20;20:774. doi: 10.1186/s13018-025-06193-1 (PMC12366400; doi:10.1186/s13018-025-06193-1)
Supplement: Supplementary file 2 — Supplementary Material 2 [file 13018_2025_6193_MOESM2_ESM.docx]

**Appendix 1**

Interview guide

Questions for participants:

- Can you tell us what you thought of being part of the trial?
- What didn’t work well, was inconvenient or unappealing in any way?
- What parts do you think worked well?
- You did 3 surveys. How would you have felt if the study went longer and you did 3 more surveys over 12 months?
- Would anything else facilitate your pain management?
- Who did you interact with about your pain medicine (e.g. in hospital, APS, ward doctors, ortho surgeon, or community e..g pharmacist, GP)
- Did you need to take other pain medicine besides what you were physically given in hospital? Did you get any extra scripts filled? How did you feel about this?
- What time point do you see as the most important. E.g. what times should we measure your pain? Which of those timepoints is the MOST important?
- What you join another trial in the future? If not, why not?

Questions for clinicians:

- What is your clinical role? (e.g. JMO, surgeon, etc). How involved with the day to day running of the trial were you?
- What has it been like taking part in this study? What parts worked well for you? What parts were troublesome or challenging?
- Were there any surprises? E.g. APS being involved when we weren’t expecting it?
- Did the trial merge well with usual practice?
- In your opinion, were the two treatment regimens adhered to (e.g. did everyone end up receiving the number of tablets they were allocated to?) If not, what went wrong?
- Would you agree to participate in a similar study again in the future?
- If not, what are the deal-breakers?
- How could we improve upon these deal-breakers?
- Is there anything else you would like to tell me about your experience taking part in this study?

**Appendix 2**

***Patients’ acceptability using 4 items Acceptability of Intervention Measure at week 1 and 2***

|  | **Week** | **Standard**  **group** | **Reduced**  **group** | **Mean difference (95% CI),**  **P-value** |  |
| --- | --- | --- | --- | --- | --- |
| Acceptability of the trial intervention (mean (SD), N) | 1 | 3.6 (0.8), 26 | 3.6 (0.7), 26 | 0.03 ( -0.40 to 0.45), p= 0.9 |  |
| Acceptability of the trial intervention (mean (SD), N) | 2 | 3.6 (0.9), 25 | 3.6 (0.8), 25 | -0.03 ( -0.51 to 0.45), p= 0.9 |  |

SD=standard deviation; N=number; 1=Completely disagree, 2=Disagree, 3=Neither agree nor disagree, 4=Agree, 5=Completely agree.

***Distribution of acceptability by the 4 item Acceptability of Intervention of the trial intervention at week 1 and 2***


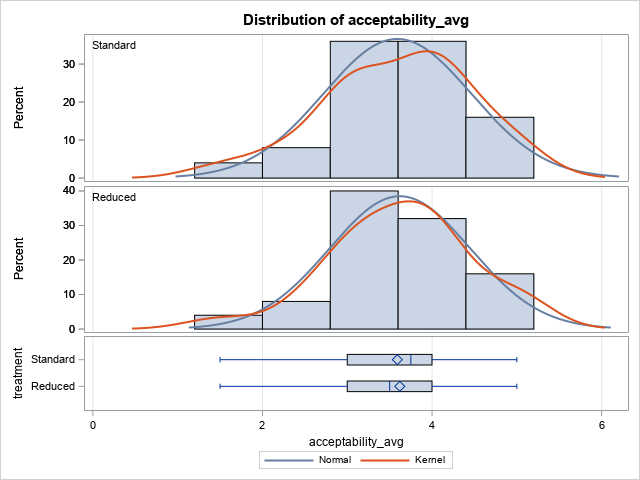


**Appendix 3**

***Specific themes and supporting quotes based on Participant interviews***

| **Specific themes** | **Supporting quotes** |
| --- | --- |
| Characteristics of intervention | |
| Participants found both the intervention and control interventions acceptable as a starting prescription. Some assumed this would be enough, while others expected to need to get more from their GP. In either case, patients found it acceptable to only be prescribed a small amount to begin with. | “I don't like using pain killers … which I don't like doing unless it's necessary.’’  “I'm pretty much off the off the Pain Killers, and you know I just take them some panicle occasionally if I feel I need it.” |
| Characteristics of individuals | |
| Some participants were hesitant to take any opioids at all (i.e. were planning to only take the non-opioid components of the trial regimens) whereas others were expecting to require the opioids. These attitudes differed based on how each person viewed their personal level of risk (e.g. belief that the serious risks are not relevant to them as they are ‘not likely’ not become addicted). | “it's repeated by all sources. So in my mind I was not worried about getting addicted.”  “I just had to get used to. It was a head thing more than anything else. It was a head thing.” |
| Many participants who joined had an altruistic attitude about participating in a trial about pain relief and opioids, and agreed that the question was highly important | “I volunteered, with no hesitation. I think it's a good thing to help people with health issues and particularly hot drugs is a big issue. And I'm happily doing it.” |
| Inner setting | |
| Participants noted some differences in communication between ward staff and study staff (e.g. some ward staff were unaware that they were on the trial and so gave inconsistent advice compared to what they were told by staff who knew about the trial). | “Well, they did not really ask me. My opinion. They just did see me in certain period of time, and it was what the doctor ordered, and take it.” |
| Outer setting | |
| Participants found the trial interventions acceptable as they believed they would be able to access their GP to receive ongoing pain medicines if required. Many took on our advice to pre-book an appointment for 2-3 days post expected discharge date. | “I had to go for a follow up with the Gp. So that that was the reason why I went to Gp, just so we could. You know, And then I did mention then that I had run out of the endone, and he wrote me a script. Yeah.” |
| Most participants found the trial interventions acceptable as they believed they would be able to access other healthcare providers to receive non-prescription pain medicines or non-pharmacological pain management options if required (e.g. pharmacists or physiotherapists) | “I mentioned that to the Physio, and and they agreed that that was the right thing to do.” |
| In rare cases, participants who lived outside of metro areas, or lived alone, were hesitant to accept the chance of being allocated to the ‘reduced’ group due to concern that they may not be able to access more opioids easily if they were required (e.g. they might have difficulty physically getting to their GP). | “It was a bit hard, because I wasn't physically near.” |
| Process | |
| Participants valued being contacted regularly by trial staff to clarify how to properly complete the diaries and surveys | “I often was thinking that the questions are structured in a way that it sometimes you don't know what to ask, to what to answer.” |
| Participants preferred a more nuanced outcome measure for pain over a simple 10-point scale due to the variable and changing nature of their pain throughout the day | “That's a good question. Because, yes, there are different times during the day when you have more pain.” |
| Participants reported that the first week after discharge is the most critical time for pain management | “particularly the first say week after discharge.” |
| Participants appreciated the options for different methods of completing surveys (e.g. some preferred online, others preferred over the phone). | “audio is more convenient, then I don't need to be near my computer at home, and so on” |
| Participants felt that the trial was missing sleep as an outcome measure. | “When I wake up I could feel the pain, and it was hard to fall asleep again.” |

GP=general practitioner. NPRS=numeric pain rating scale

***Specific themes and supporting quotes based on Clinician’ interview***

| **Specific themes** | **Supporting quotes** |
| --- | --- |
| Outer setting | |
| Staff reported that inclusion in the trial was not feasible for participants that were being discharged to rehabilitation or other hospitals as we could not control their discharge prescriptions, and so they were not randomized despite having been willing to join the trial. | “changing their medication or whether they are going to a rehab hospital.”  “And sometimes they we don't even know that they've changed their medication until later on.” |
| Inner setting | |
| In some cases, the Acute Pain Service got involved in the prescribing pain medication for discharge during the participant’s inpatient stay and did not necessarily stick with the trial protocol. These patients were therefore not randomized. | “you know the timing or the strength of their pain, relief, and talk through all the kind of clinical situation there. So that would be kind of standard practice of how the Pain Service would be involved. So I guess we did just assume they would be involved.” |
| Process |  |
| Junior Medical Officers from the orthopaedic ward were tasked with screening potential participants in the pre-admission clinic, however on some occasions they were too busy to go down for screening. It may be more feasible to have staff situated in the pre-admission clinic do the screening. | “I think the main thing is when we recruit we couldn't sometimes couldn't get the JMO to come over because they are so busy”.  “That's tricky is just trying to fit that time in when it is quite time sensitive amongst our other busy days, so I don't know what my suggestion to an alternative would be there.” |
| Keeping up-to-date on each participant’s planned date of discharge was challenging as this can change at short notice. If a patient was discharged suddenly, trial staff may not have had time to randomize them. | “It's hard for us to catch them as well to to do the follow up, or whatever is needed on that day.” |

AOANJRR=Australian Orthopaedic association national joint replacement registry. eMR=electronic medical records. JMO=junior medical officer.
